# Supplementary material for: MSNGO: multi-species protein function annotation based on 3D protein structure and network propagation
Source: Bioinformatics. 2025 May 6;41(5):btaf285. doi: 10.1093/bioinformatics/btaf285 (PMC12122197; doi:10.1093/bioinformatics/btaf285)
Supplement: btaf285_Supplementary_Data [file btaf285_supplementary_data.docx]

# Supplemental Data

## Data

**Table S1.** Dataset statistic of different species.

| **Species** | **Train** | | | **Valid** | | | **Test** | | |
| --- | --- | --- | --- | --- | --- | --- | --- | --- | --- |
|  | **BPO** | **MFO** | **CCO** | **BPO** | **MFO** | **CCO** | **BPO** | **MFO** | **CCO** |
| Homo sapiens | 13453 | 13085 | 13711 | 726 | 619 | 668 | 537 | 535 | 615 |
| Mus musculus | 13617 | 12505 | 13037 | 995 | 899 | 934 | 850 | 887 | 786 |
| Saccharomyces cerevisiae | 4828 | 4144 | 5180 | 7 | 8 | 6 | 5 | 4 | 14 |
| Drosophila melanogaster | 7046 | 5950 | 6849 | 474 | 528 | 522 | 362 | 433 | 361 |
| Zea mays | 4432 | 4153 | 4141 | 805 | 1131 | 1199 | 1228 | 1726 | 1321 |
| Danio rerio | 7557 | 6303 | 6749 | 1094 | 954 | 1155 | 693 | 734 | 671 |
| Dictyostelium discoideum | 3186 | 2850 | 3196 | 387 | 444 | 520 | 353 | 471 | 412 |
| Schizosaccharomyces pombe | 3370 | 3126 | 4469 | 31 | 26 | 39 | 36 | 41 | 33 |
| Caenorhabditis elegans | 5669 | 4843 | 5355 | 658 | 651 | 693 | 534 | 776 | 571 |
| Arabidopsis thaliana | 7935 | 7080 | 8405 | 259 | 287 | 1167 | 87 | 86 | 87 |
| Rattus norvegicus | 10652 | 9390 | 10088 | 1441 | 1237 | 1536 | 987 | 1051 | 1016 |
| Escherichia coli K-12 | 2775 | 2634 | 2441 | 17 | 19 | 22 | 13 | 14 | 5 |
| Haemophilus influenzae Rd | 577 | 582 | 505 | 49 | 60 | 41 | 55 | 72 | 55 |
| Total | 85097 | 76645 | 84126 | 6943 | 6863 | 8502 | 5740 | 6830 | 5947 |

## 2. Evaluation Metrics

（1）$F_{max}$ is the highest F1−score among all thresholds $\tau$, which takes into account both precision and recall. The formula is as follows:

$F_{max}=\max_{\tau} \left\{ \frac{2\cdot avgPr(\tau)\cdot avgRc(\tau)}{avgPr(\tau)+avgRc(\tau)} \right\}$ (S2-1)

where $avgPr(\tau)$ and $avgRc(\tau)$ represent the average precision and average recall at threshold $\tau$, respectively. Their calculation methods are as follows:

$avgPr(\tau)=\frac{1}{N}\sum_{i=1}^{N(\tau)} \frac{|P_{i}(\tau)\cap T_{i}|}{|P_{i}(\tau)|}$ (S2-2)

$avgRc(\tau)=\frac{1}{N}\sum_{i=1}^{N} \frac{|P_{i}(\tau)\cap T_{i}|}{|T_{i}|}$ (S2-3)

where $N$ represents the number of protein samples, $N(\tau)$ represents the number of protein samples with at least one predicted score not less than $\tau$. $P_{i}(\tau)$ is the set of functional labels for the 𝑖-th protein with predicted scores not less than $\tau$, $T_{i}$ is the set of true labels for the 𝑖-th protein, and $|\cdot|$ denotes the number of elements in a set.

（2）AUPR is the area under the precision-recall curve, We use the above $avgPr(\tau)$ and $avgRc(\tau)$ to draw the PR curve to get the AUPR value. AUPR is suitable for evaluating the imbalance of classification samples and can measure the model's ability to balance recall and precision.

（3）$S_{min}$ is calculated based on the Information Content (IC) of the label categories. The formula for IC is as follows:

$IC(c)=-log(P\left( c \right|Fa\left( c \right)))$ (S2-4)

where $IC(c)$ represents the IC value of label $c$, $Fa(c)$ denotes the set of parent labels of $c$, and $P(\cdot)$ represents the conditional probability. $S_{min}$ is calculated from IC as follows:

$S_{min}=\min_{\tau} \sqrt{{ru(\tau)}^{2}+{mi(\tau)}^{2}}$ (S2-5)

$u\left( \tau\right)=\frac{1}{N}\sum_{i=1}^{N} \sum_{c\in T_{i}-P_{i}\left( \tau\right)} IC(c)$ (S2-6)

$mi\left( \tau\right)=\frac{1}{N}\sum_{i=1}^{N} \sum_{c\in P_{i}\left( \tau\right)-T_{i}} IC(c)$ (S2-7)

where $ru\left( \tau\right)$ represents the average remaining uncertainty at threshold $\tau$, and $mi\left( \tau\right)$ is the average misinformation at threshold $\tau$.

## 3. Weighted Evaluation Metrics

We did find that some methods use IC (Information Content) as the weight of the label, which can better reflect the model's prediction ability for certain specific functions. To this end, we adopted your suggestion and re-evaluated the prediction results of baselines and MSNGO using weighted Fmax and AUPR. The calculation formula is as follows:

$wF_{max}=\max_{\tau} \left\{ \frac{2\cdot wPr(\tau)\cdot wRc(\tau)}{wPr(\tau)+wRc(\tau)} \right\}$ (S3-1)

$wPr(\tau)=\frac{1}{N}\sum_{i=1}^{N(\tau)} \frac{\sum_{q}^{Q} P_{i,q}\left( \tau\right)\cdot T_{i,q}\cdot IC(q)}{\sum_{q}^{Q_{i}} P_{i,q}\left( \tau\right)\cdot IC(q)}$ (S3-2)

$wRc(\tau)=\frac{1}{N}\sum_{i=1}^{N} \frac{\sum_{q}^{Q} P_{i,q}\left( \tau\right)\cdot T_{i,q}\cdot IC(q)}{\sum_{q}^{Q_{i}} T_{i,q}\cdot IC(q)}$ (S3-3)

where $wF_{max}$ represents the Fmax value after IC weighting, $wPr(\tau)$ and $wRc(\tau)$ represent the weighted precision and weighted regression rate under the threshold τ, respectively, and wAUPR is also calculated using $wPr(\tau)$ and $wRc(\tau)$. N represents the number of protein samples, $N(\tau)$ represents the number of protein samples with at least one predicted score not less than $\tau$. $Q_{i}$ represents the label set of protein i. $P_{i,q}\left( \tau\right)\in\{0,1\}$ represents whether the predicted value of protein i on label q is greater than $\tau$. When its predicted value is greater than $\tau$, $P_{i,q}\left( \tau\right)$ takes 1, otherwise it takes 0. $T_{i,q}$ represents the true value of protein i on label q, and $IC(q)$ is the IC value mentioned in the above metrics.

## 4. Evidence Code

To ensure the reliability of the function labels, we only retained protein annotations that were manually curated by experts and experimentally validated, with evidence codes EXP, IDA, IPI, IMP, IGI, IEP, HTP, HDA, HMP, HGI, HEP, IBA, IBD, IKR, and IRD.

It is worth noting that our dataset incorporates phylogenetically-inferred annotations, which serve to enrich the GO terms of proteins with limited annotations and not affect our experimental results. To verify this point of view, we specially constructed a dataset called dataset-limited that only contains EXP, IDA, IPI, IMP, IGI, and IEP. Except for the different evidence codes, the data processing of this dataset is consistent with the original dataset. The distribution of the dataset-limited is shown in Table S2. There are 19266 bp labels, 6406 mf labels, and 2632 cc labels after sorting.

**Table S2.** The statistical results of the dataset-limited, which exclusively includes evidence codes EXP, IDA, IPI, IMP, IGI, and IEP.

| **ontology** | **Train** | **Valid** | **Test** |
| --- | --- | --- | --- |
| BPO | 51028 | 1302 | 694 |
| MFO | 41872 | 1048 | 424 |
| CCO | 40859 | 1009 | 1238 |

To comprehensively evaluate the model performance, we replicated the comparative experiments of MSNGO and baselines on the dataset-limited, with the results detailed in Table S3. In comparison to the findings presented in Table 2, a marked decline is observed across all metrics. However, the performance differences between the methods remain relatively stable.

**Table S3.** Comparative experimental results on data-limited

| **Model** | **BPO** | | | **MFO** | | | **CCO** | | |
| --- | --- | --- | --- | --- | --- | --- | --- | --- | --- |
|  | **Fmax** | **Smin** | **AUPR** | **Fmax** | **Smin** | **AUPR** | **Fmax** | **Smin** | **AUPR** |
| SPROF-GO | 0.3554 | 21.8135 | 0.3066 | 0.3554 | **5.2905** | **0.5880** | **0.6811** | 7.7705 | 0.6997 |
| DeepGraphGO+ESM-2 | 0.2470 | 25.1326 | 0.1712 | 0.2960 | 7.7338 | 0.1646 | 0.6088 | 8.9108 | 0.6587 |
| PSPGO+ESM-2 | 0.3534 | 22.3524 | 0.2842 | 0.4805 | 6.6896 | 0.4675 | 0.6196 | 8.1288 | 0.6817 |
| MSNGO+ESM-2 | **0.3907** | **21.4758** | **0.3248** | **0.5724** | 5.9433 | 0.5533 | 0.6505 | **7.5620** | **0.7173** |
| DeepGraphGO+Interproscan | 0.4187 | 21.8872 | **0.3517** | 0.6448 | 5.0653 | 0.6099 | **0.6723** | 7.6898 | 0.6711 |
| PSPGO+Interproscan | 0.4132 | 20.5500 | 0.3509 | **0.6540** | **4.7860** | 0.6146 | 0.6476 | 7.9211 | 0.7142 |
| MSNGO+Interproscan | **0.4194** | **20.2551** | 0.3464 | 0.6369 | 4.9906 | **0.6196** | 0.6614 | **7.4196** | **0.7359** |

In the comparative experiments based on ESM-2 features, MSNGO shows a significant advantage, achieving the best results in most indicators, with only three indicators at suboptimal levels. In the experiments based on Interproscan, the three methods have their own strengths, and the distribution of the best indicators is relatively balanced, but MSNGO still dominate, with only two indicators not reaching the optimal or suboptimal level.

Although we removed the phylogenetic GO annotations, we still observed similar trends to the results of the original dataset: When using Interproscan as sequence features, the performance differences among the three comparative methods are small. However, when employing features extracted by ESM-2, DeepGraphGO and PSPGO exhibit a significant decline in performance, while MSNGO demonstrates excellent stability. This finding suggests that although the lack of phylogenetic annotations leads to a significant reduction in metrics such as Fmax, it does not compromise the validity of the experimental conclusions. The MSNGO method shows outstanding classification performance and robustness in the comparative experiment, confirming the advantages of this method.

## 5. Experimental Results

**Table S4.** Comparative experimental results after using weighted Fmax and weighted AUPR.

| **Model** | **BPO** | | | **MFO** | | | **CCO** | | |
| --- | --- | --- | --- | --- | --- | --- | --- | --- | --- |
|  | **wFmax** | **Smin** | **wAUPR** | **wFmax** | **Smin** | **wAUPR** | **wFmax** | **Smin** | **wAUPR** |
| SPROF-GO | 0.4407 | 10.1893 | 0.3800 | 0.7315 | 2.7099 | 0.7487 | 0.7081 | 3.2319 | 0.7577 |
| DeepGraphGO+ESM-2 | 0.1966 | 16.8778 | 0.1193 | 0.2034 | 9.5505 | 0.1003 | 0.4471 | 4.7173 | 0.3773 |
| PSPGO+ESM-2 | 0.5770 | 8.0511 | 0.6017 | 0.5891 | 4.7816 | 0.5862 | 0.5570 | 4.1838 | 0.5495 |
| MSNGO+ESM-2 | **0.6917** | **5.8527** | **0.7200** | **0.7823** | **2.4884** | **0.8359** | **0.7184** | **2.8478** | **0.7658** |

**Table S5.** Comparative experiment with the single-species model Struct2GO on two single-species data.

| **species** | **Method** | **BPO** | | **MFO** | | **CCO** | |
| --- | --- | --- | --- | --- | --- | --- | --- |
|  |  | **Fmax** | **AUPR** | **Fmax** | **AUPR** | **Fmax** | **AUPR** |
| Homo sapiens | Struct2GO | 0.4660 | 0.5212 | 0.7017 | 0.7944 | 0.6437 | 0.7510 |
|  | MSNGO | **0.8328** | **0.7312** | **0.9418** | **0.9618** | **0.9731** | **0.9173** |
| Saccharomyces cerevisiae | Struct2GO | 0.4113 | 0.3634 | 0.6000 | 0.4069 | 0.6556 | 0.5462 |
|  | MSNGO | **0.6097** | **0.6231** | **0.7709** | **0.7649** | **0.8946** | **0.9048** |

Homo sapiens comes from the test set provided by Struct2GO, and is predicted directly using the trained MSNGO and Struct2GO model files respectively. Saccharomyces cerevisiae comes from the dataset provided by MSNGO. In order to use Struct2GO prediction, we retrained a model for Struct2GO using this dataset.

**Table S6.** The evaluation results when the graph pooling rate k is 0.25, 0.5, 0.75 and 0.8 respectively

| **k value** | **wFmax** | **Smin** | **wAUPR** |
| --- | --- | --- | --- |
| 0.25 | 0.7642 | 2.3637 | 0.8337 |
| 0.5 | 0.7770 | **2.3222** | 0.8294 |
| 0.75 | **0.7823** | 2.4884 | **0.8359** |
| 0.8 | 0.7467 | 2.6329 | 0.8160 |

**Table S7.** Comparative experimental results between DPFunc and MSNGO on Saccharomyces cerevisiae

| **Model** | **BPO** | | **MFO** | | **CCO** | |
| --- | --- | --- | --- | --- | --- | --- |
|  | **Fmax** | **AUPR** | **Fmax** | **AUPR** | **Fmax** | **AUPR** |
| DPFunc | **0.7429** | **0.7608** | 0.7238 | 0.7607 | 0.8246 | 0.8775 |
| MSNGO | 0.6097 | 0.6231 | **0.7709** | **0.7649** | **0.8946** | **0.9048** |

DPFunc significantly outperformed MSNGO in the Biological Process (BP) category by 14%, while MSNGO showed slightly better performance than DPFunc in both Molecular Function (MF) and Cellular Component (CC) categories. This discrepancy may be attributed to the excessive number of GO terms in BP. MSNGO retains phylogenetically informative GO annotations, aiming to provide proteins with richer functional information, which benefits both MF and CC predictions. However, the BP category contains nearly 20,000 GO terms, creating substantial classification pressure and leading to suboptimal performance for MSNGO in this category.
